# Extracellular vesicle-mediated delivery of genetic material for transformation and CRISPR/Cas9-based gene editing in *Pneumocystis murina*

**DOI:** 10.1128/mbio.01825-25

**Published:** 2025-09-23

**Authors:** Steven G. Sayson, Alan Ashbaugh, Lillian C. Bauer, A. George Smulian

**Affiliations:** 1 Department of Internal Medicine, University of Cincinnati College of Medicine 12303, Cincinnati, Ohio, USA; 2 The Veterans Affairs Medical Center, Cincinnati, Ohio, USA; 3 Department of Pharmacology, Physiology and Neurobiology, University of Cincinnati College of Medicine 12303, Cincinnati, Ohio, USA; 4 Department of Biology, University of Kentucky College of Arts and Sciences 123977, Lexington, Kentucky, USA; IMBB-FORTH, Heraklion, Greece

**Keywords:** *Pneumocystis*, extracellular vesicles, homology-directed repair, CRISPR/Cas9, genetic manipulation, functional genomics, mycology, antifungal resistance

## Abstract

*Pneumocystis* species are obligate fungal pathogens that cause severe pneumonia, particularly in immunocompromised individuals. The absence of robust genetic manipulation tools has impeded our mechanistic understanding of *Pneumocystis* biology and the development of novel therapeutic strategies. Herein, we describe a novel method for the stable transformation and CRISPR/Cas9-mediated genetic editing of *Pneumocystis murina* utilizing extracellular vesicles (EVs) as a delivery vehicle. Building upon our prior investigations demonstrating EV-mediated delivery of exogenous material to *Pneumocystis*, we engineered mouse lung EVs to deliver plasmid DNA encoding reporter genes and CRISPR/Cas9 components. Our initial findings demonstrated successful *in vitro* transformation and subsequent expression of *mNeonGreen* and *Dhps^ARS^* in *P. murina* organisms. Subsequently, we established stable *in vivo* expression of *mNeonGreen* in mice infected with transformed *P. murina* for a duration of up to 5 weeks. Furthermore, we designed and validated a CRISPR/Cas9 system targeting the *P. murina Dhps* gene, confirming DNA cleavage efficiency *in vitro*. Ultimately, we achieved successful *in vivo* CRISPR/Cas9-mediated homologous recombination, precisely introducing a *Dhps^ARS^* mutation into the *P. murina* genome, which was confirmed by Sanger sequencing across all tested animals. Here, we establish a foundational methodology for genetic manipulation in *Pneumocystis*, thereby opening avenues for functional genomics, drug target validation, and the generation of genetically modified strains for advanced research and potential therapeutic applications.

**IMPORTANCE:**

*Pneumocystis* species are obligate fungal pathogens and major causes of pneumonia in immunocompromised individuals. However, their strict dependence on the mammalian lung environment has precluded the development of genetic manipulation systems, limiting our ability to interrogate gene function, study antifungal resistance mechanisms, or validate therapeutic targets. Here, we report the first successful approach for stable transformation and CRISPR/Cas9-based genome editing of *Pneumocystis murina*, achieved through *in vivo* delivery of engineered extracellular vesicles containing plasmid DNA and encoding CRISPR/Cas9 components. We demonstrate sustained transgene expression and precise modification of the *dhps* locus via homology-directed repair. This modular, scalable platform overcomes a long-standing barrier in the field and establishes a foundation for functional genomics in *Pneumocystis* and other obligate, host-adapted microbes.

## INTRODUCTION

*Pneumocystis jirovecii* pneumonia (PjP) remains a critical cause of morbidity and mortality in immunocompromised individuals, including those with HIV/AIDS, hematologic malignancies, or organ transplants receiving immunosuppressive therapy (1–4). Despite significant clinical relevance, critical aspects of the obligate fungal pathogen *Pneumocystis* remain poorly characterized. Key gaps, including its biology, pathogenesis, and antifolate drug resistance, remain primarily due to the persistent inability to culture these fungi *in vitro* (5). Unlike other fungi that readily grow in defined media, *Pneumocystis* species are obligate biotrophs, requiring a mammalian host to replicate (6). The lack of a long-term culture system severely limits functional genetic studies and the exploration of novel therapeutic targets.

A significant clinical challenge associated with *Pneumocystis* treatment is resistance to the antifolate drug combination trimethoprim-sulfamethoxazole (TMP-SMX), which remains the first-line therapeutic option (7, 8). Mutations in the dihydropteroate synthase (*Dhps*) gene, notably at codons 55 and 57, confer SMX resistance, complicating therapeutic management and necessitating novel methods to dissect resistance mechanisms and validate alternative therapeutic targets (9–13). These mutations result in an amino acid change from TRP (wild type) to ARS (SMX-resistant).

Furthermore, *Pneumocystis* genomes feature numerous major surface glycoprotein (*Msg*) genes arranged in tandem repeats near telomeric regions (14–16). These *Msg* genes are hypothesized to undergo frequent genetic recombination, enabling antigenic variation through differential expression of individual *Msg* variants (17, 18). The extensive and rapid genetic rearrangements observed in *Pneumocystis* imply a highly efficient DNA recombination system, suggesting that targeted genome editing via homologous recombination (HR) may be inherently feasible.

Recently, extracellular vesicles (EVs) have emerged as critical mediators of intercellular communication, transferring proteins, lipids, and nucleic acids between cells (19). Our prior work demonstrated that *Pneumocystis* organisms actively internalize host-derived EVs, presumably as a nutrient acquisition mechanism (20). We reasoned that this natural EV uptake pathway could be exploited to deliver genetic constructs, facilitating genetic manipulation of *Pneumocystis in vivo*.

CRISPR/Cas9-mediated genome editing technology has transformed genetic engineering, allowing targeted disruption, insertion, or modification of specific genomic loci (21, 22). Adapting this technology for *Pneumocystis* would significantly enhance our ability to probe gene function, dissect resistance mechanisms, and identify novel therapeutic targets.

In the present investigation, we aimed to develop a comprehensive methodology for genetic manipulation in *Pneumocystis murina*, encompassing two distinct, yet complementary approaches: stable plasmid-based transformation for gene overexpression and precise CRISPR/Cas9-mediated gene editing for targeted genomic modifications. We hypothesized that engineered mouse lung EVs could effectively deliver plasmid constructs to *P. murina*, enabling stable expression of foreign genes and facilitating targeted genomic modifications. Herein, we present compelling data demonstrating successful *in vitro* transformation, the establishment of *in vivo* persistence of transformed organisms, and the achievement of precise CRISPR/Cas9-mediated homologous recombination at the *Dhps* locus, a critical gene implicated in antifolate drug resistance. This work represents a significant leap forward in *Pneumocystis* research, providing essential tools that will facilitate future functional genomic studies, enable the creation of genetically modified strains, and ultimately contribute to the development of more effective therapeutic strategies against PjP.

## RESULTS

### EV uptake by *P. murina*

**Fig 1:**
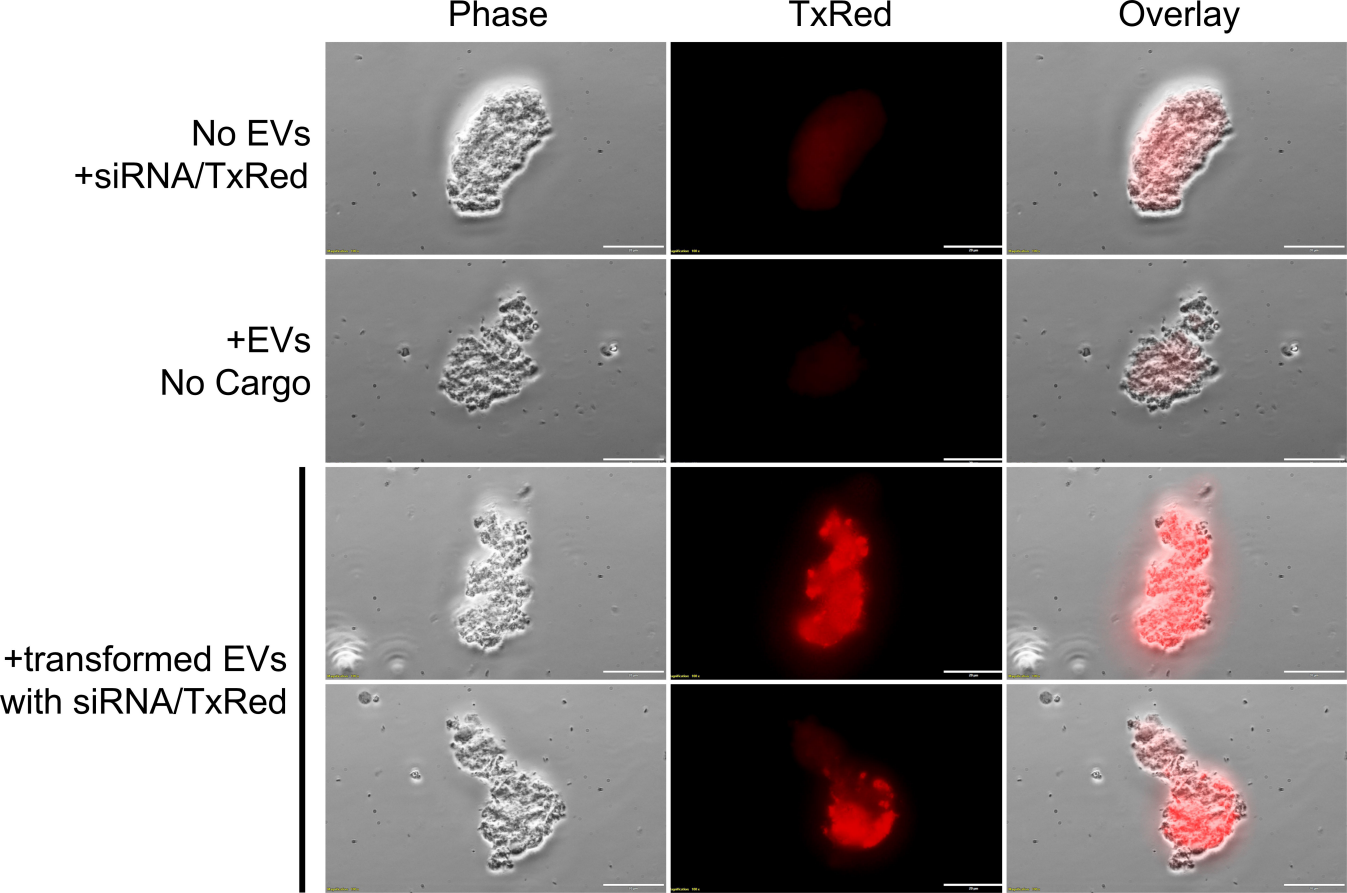
*Pneumocystis murina* uptake of BALF EVs containing exogenous nucleotides. *P. murina* was treated with TxRed-conjugated siRNA (top row), EVs alone (middle row), or EVs loaded with siRNA-TxRed (bottom rows) for 16 h. Scale bars, 20 µm.

Our previous work demonstrated that *Pneumocystis carinii* actively uptakes native host lung EVs labeled with the lipophilic dye PKH26, suggesting a mechanism for nutrient acquisition from the host (20). Building on this, we conducted an initial feasibility study to determine if *P. murina* could internalize EVs containing exogenous nucleotides. EVs transformed with TxRed-labeled siRNA were co-cultured with *P. murina* for 24 h. Fluorescent microscopy analysis (Fig. 1) demonstrated clear uptake of the TxRed signal by *P. murina* organisms. In the control group, where *P. murina* was co-cultured with siRNA-TxRed but no EVs, no TxRed signal was observed within the organisms. Similarly, *P. murina* co-cultured with EVs containing no cargo also showed no TxRed signal. In contrast, *P. murina* co-cultured with EVs loaded with siRNA-TxRed exhibited distinct punctate TxRed fluorescence localized within the fungal cells, indicating successful internalization of the EV cargo. This confirms that *P. murina* can actively take up EVs and their contents, establishing a foundational step for EV-mediated genetic delivery.

### Successful EV-mediated gene delivery and expression in *P. murina*

**Fig 2:**
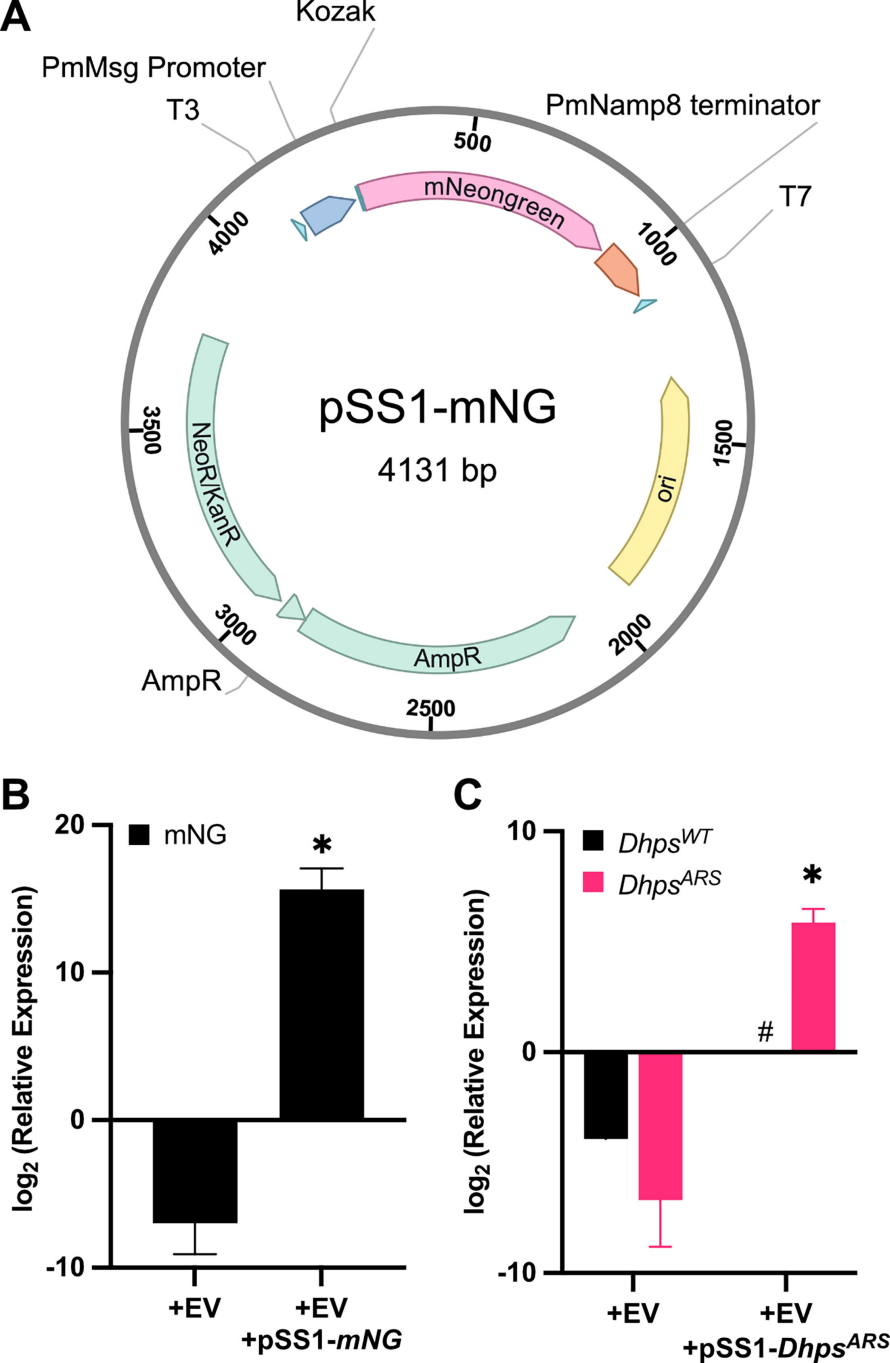
*Pneumocystis murina* cultured *in vitro* transcribes plasmid-encoded genes following delivery by extracellular vesicles. (**A**) Plasmid map of pSS1-mNG, which encodes *mNeonGreen* driven by the Pm *Msg* promoter, and a Namp8 terminator. *P. murina* (1 × 10^6^ organisms) was treated with EVs (2 µg protein equivalent) loaded with either pSS1-*mNeonGreen* (mNG) or pSS1-*Dhps*^*ARS*^ for 16 h. Total RNA was extracted from the cells and synthesized to cDNA. Reverse transcription quantitative PCR (RT-qPCR) was performed, and relative expression was calculated using the 2^−ΔCt^ method, normalized to large subunit (LSU). Plasmid-transformed (**B**) *mNeonGreen* and (**C**) *Dhps*^*ARS*^ groups (*n* = 9) showed significant transcriptional activity, confirming successful expression of the plasmid-delivered genes. Native *Dhps*^*WT*^ was notably absent in plasmid-transformed *P. murina*. *t*-test; ∗, *P* < 0.05 against control. #, unable to perform comparison due to the lack of detectable expression.

The initial pSS1 plasmid (Fig. 2A) was constructed, incorporating an expression cassette with a flanking *P. murina Msg* promoter and *PmNamp8* terminator to ensure the expression of introduced genes within the fungal host. *P. murina* codon-optimized *mNeonGreen* or *Dhps^ARS^* were cloned into the coding region.

*P. murina* organisms co-cultured with EVs containing either pSS1-*mNeonGreen* or pSS1-*Dhps^ARS^* demonstrated successful gene delivery and subsequent transcriptional activity *in vitro* after 24 h. Relative to *P. murina* exposed to empty EVs (control), the *mNeonGreen* and *Dhps^ARS^* groups showed strong transcriptional activity, with average 2^−ΔCt^ values of 51,333 and 58.95, respectively. These correspond to log2-transformed values of approximately 15.7 and 5.9, confirming successful expression of the plasmid-delivered genes (Fig. 2B and C). Interestingly, endogenous *Dhps^WT^* transcription was not detected in pSS-*Dhps*^*ARS*^ transformed Pm, suggesting a feedback mechanism regulating gene expression. Western blot analysis did not detect mNeonGreen protein expression (data not shown), likely due to the lack of a long-term culture system. Under these conditions, *P. murina* is unable to maintain cellular homeostasis, thereby likely limiting its capacity for efficient protein translation.

This initial *in vitro* success established the feasibility of EV-mediated gene delivery for *Pneumocystis* and foundational proof of concept for the entire study. It demonstrates that EVs can indeed deliver genetic material to *P. murina* and, crucially, that this material is transcriptionally active within the fungal cells. Without this initial success *in vitro*, the more complex *in vivo* experiments and CRISPR applications would not be feasible.

### Development of a stable *in vivo* transformation system

**Fig 3:**
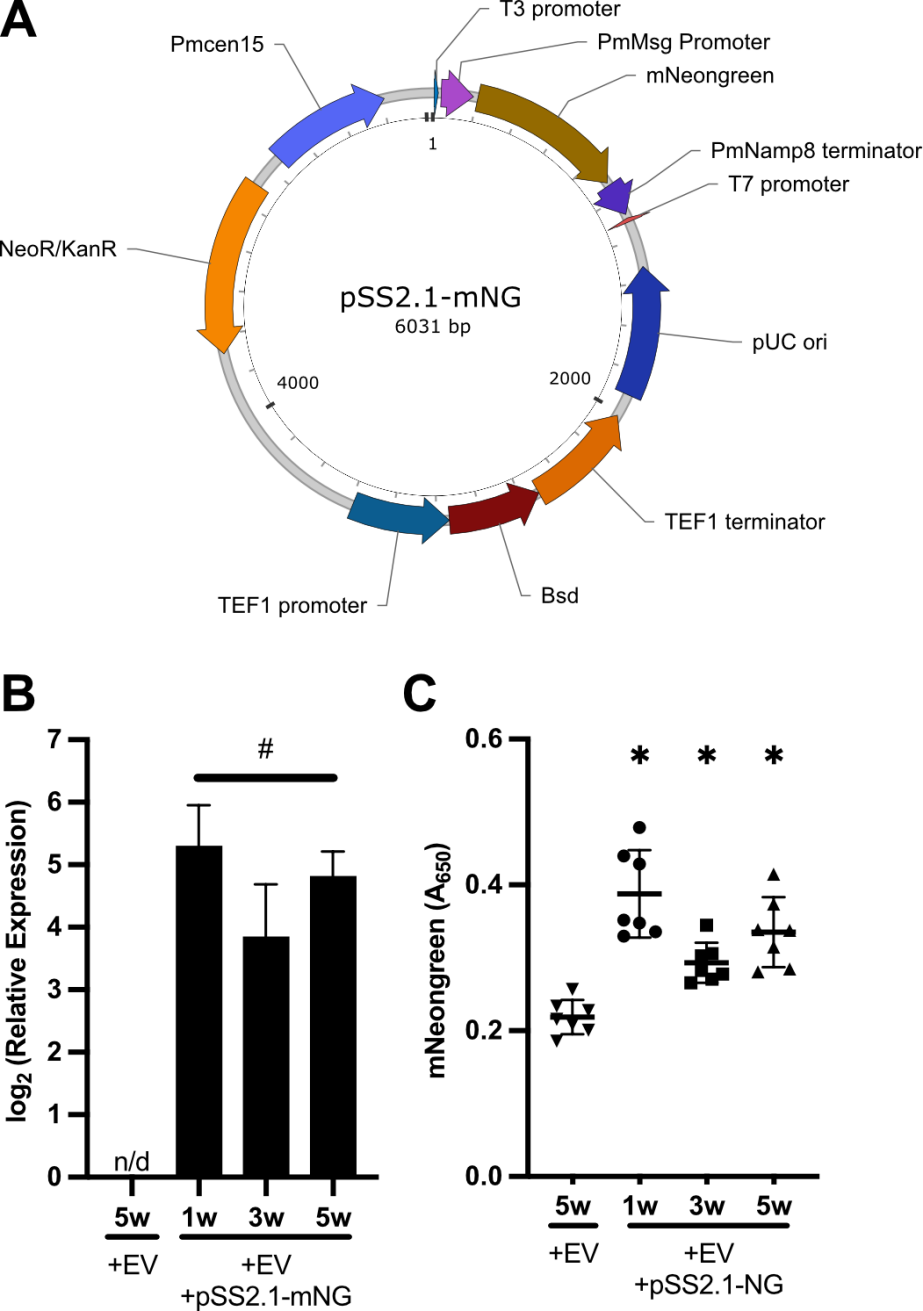
*Pneumocystis murina* expresses mNeonGreen *in vivo* following *in vitro* extracellular vesicle-mediated gene delivery. (**A**) Plasmid map of pSS2.1-*mNG*, which includes a second expression cassette encoding blasticidin S deaminase (*Bsd*) and *PmCen15*, a truncated centromeric sequence. *P. murina* (1 × 10⁶ organisms) was incubated for 16 h with extracellular vesicles (2 µg protein equivalent) either lacking cargo or loaded with pSS2.1-mNeonGreen (*mNG*). Total RNA was extracted, synthesized to cDNA, and RT-qPCR was performed. Relative expression was calculated using the 2^−ΔCt^ method, normalized to LSU. (**B**) Pm displayed sustained expression of *mNeonGreen* mRNA expression after 1 week, 3 weeks, and 5 weeks post-inoculation (*n* = 3). (**C**) mNeonGreen protein was detected in *P. murina* lysates by enzyme-linked immunosorbent assay (ELISA) after 1 week, 3 weeks, and 5 weeks post-inoculation (*n* = 3). Analysis of variance (ANOVA) followed by Sidak’s multiple comparisons *post hoc* test; n/d, no detectable expression; #, unable to perform comparison due to the lack of detectable expression; ∗, *P* < 0.05 against control.

To enable stable and long-term gene expression within the host, the pSS2.1 plasmid (Fig. 3A) was engineered. This improved construct incorporates a blasticidin resistance (blasticidin S deaminase [*Bsd*]) gene for selective pressure and, critically, a truncated centromeric region, *PmCen15* (23). This centromeric region was incorporated to ensure stable maintenance and accurate segregation of the plasmid within *P. murina* cells *in vivo*, mimicking chromosomal behavior and preventing plasmid loss, a common challenge with traditional episomal plasmids in eukaryotic systems in the absence of continuous selective pressure.

Following *in vitro* blasticidin selection of *P. murina* transformed with pSS2.1-*mNeonGreen*-containing EVs and subsequent infection of mice, *mNeonGreen* transcript expression was detected by reverse transcription quantitative PCR (RT-qPCR) and subsequent agarose gel electrophoresis (Fig. 3B). While mNeonGreen was indetectable by immunofluorescence, likely due to low expression, mNeongreen protein expression was successfully detected from Pm lysates using enzyme-linked immunosorbent assay (ELISA) (Fig. 3C). Both mNeonGreen mRNA and protein expression were sustained in lung samples collected at 1 week, 3 weeks, and 5 weeks post-infection. This sustained expression over an extended period clearly indicates successful long-term maintenance and active expression of the pSS2.1 plasmid within the *P. murina* population residing in the host lung environment.

### CRISPR/Cas9 system enables targeted genetic editing in *P. murina*

**Fig 4:**
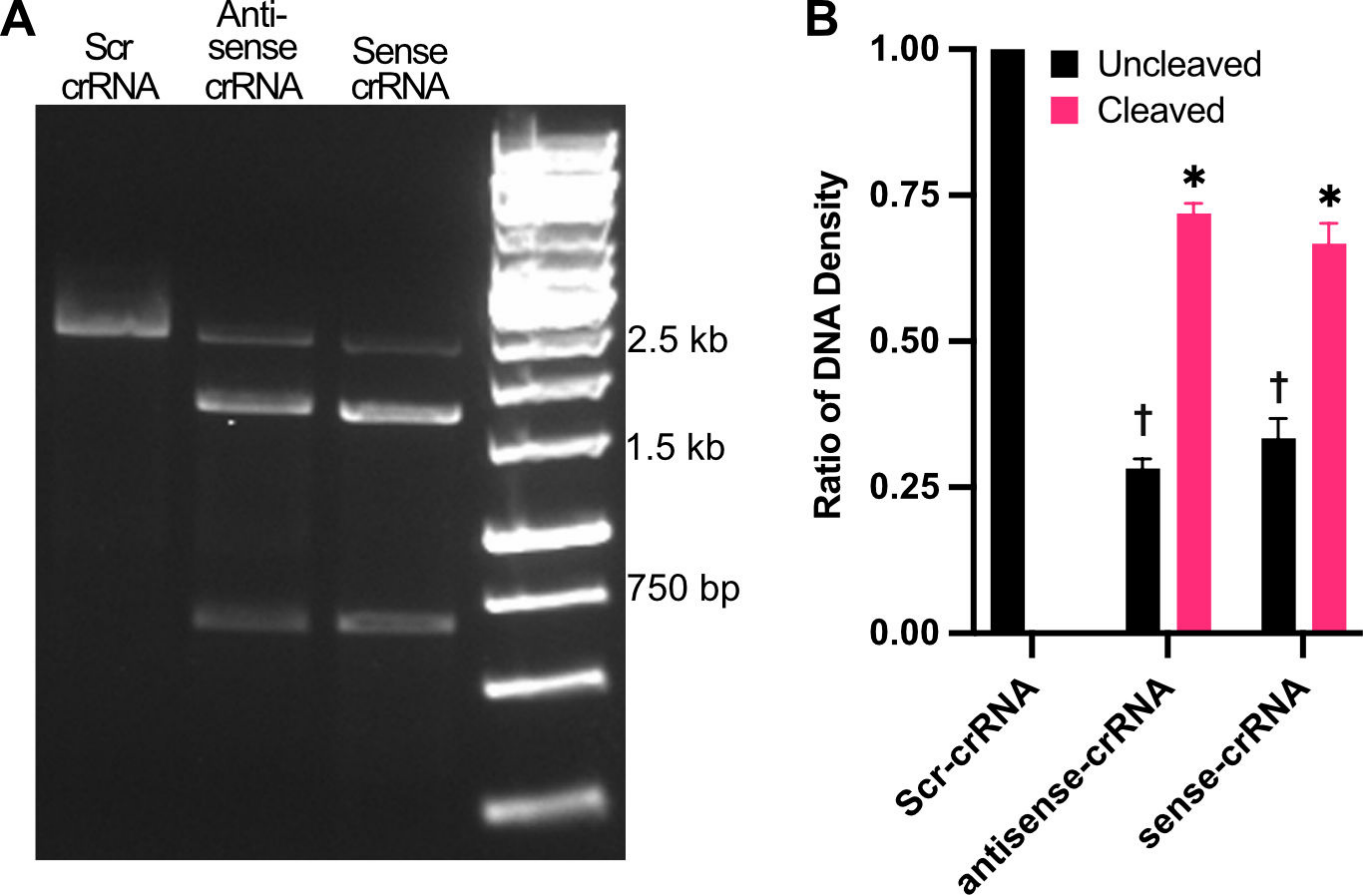
CRISPR RNA (crRNA) sequences mediate efficient cleavage of *Dhps* amplicons *in vitro*. A full-length *Dhps* amplicon was generated from *P. murina* genomic DNA. Annealed crRNA and tracrRNA were complexed with *Streptococcus pyogenes* Cas9 nuclease to form ribonucleoprotein (RNP) complexes, which were then incubated with the *Dhps* amplicons for 1 h. (**A**) Agarose gel electrophoresis showed cleavage of *Dhps* by both sense and antisense RNPs, yielding two fragments of ~1,560 bp and ~660 bp, as predicted. Scramble (scr) RNPs did not induce cleavage. (**B**) Densitometric analysis confirmed significantly increased cleavage by sense and antisense RNPs compared to scr-RNPs (*n* = 3). ANOVA followed by Sidak’s multiple comparisons *post hoc* test; †, *P* < 0.05 vs uncleaved control; *, *P* < 0.05 vs cleaved control.

Two distinct sets of crRNAs designed to target the *Dhps* gene were validated through *in vitro* cleavage assays. Agarose gel electrophoresis and subsequent densitometry analysis (Fig. 4A and B) confirmed efficient cleavage of the *Dhps* target DNA, demonstrating the functionality of the designed crRNAs and the Cas9 enzyme in a cell-free system. This *in vitro* validation was a prerequisite for proceeding with *in vivo* applications.

**Fig 5:**
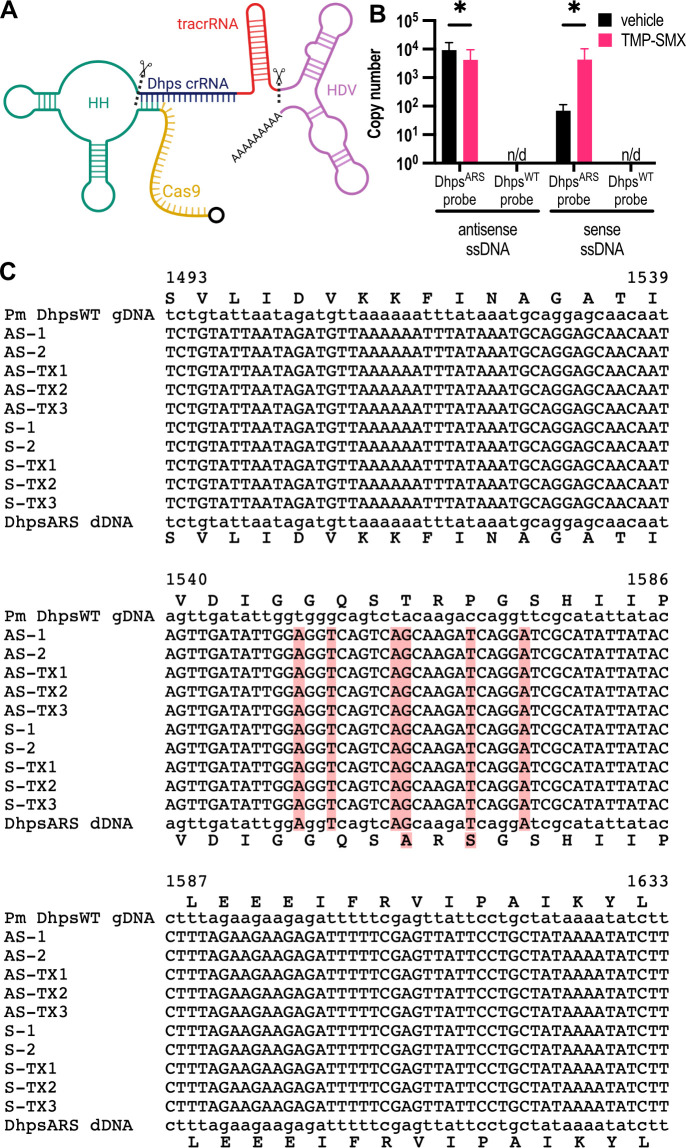
pSS2.1 effectively delivers Cas9 and gRNA for genetic editing of the *Dhps* locus. (**A**) Schematic of the *Cas9*-gRNA expression cassette, which encodes *Cas9* and a guide RNA flanked by a hammerhead (HH) ribozyme and a hepatitis delta virus (HDV) ribozyme. Created in BioRender (S. Sayson, 2025, https://BioRender.com/dp0twrz). (**B**) After 5 weeks of infection and 2 weeks of treatment, quantitative PCR detects *Dhps^ARS^* in DNA extracted from *P. murina* in both antisense-ssDNA-treated and sense-ssDNA-treated groups (*n* = 5). In the same *P. murina* populations, *Dhps^WT^* was not detected. ANOVA followed by Sidak’s multiple comparisons *post hoc* test; *, *P* < 0.05 (**C**) Alignment of the *Dhps* [1493–1633] regions from untreated organisms (Pm DhpsWT gDNA) was compared to *P. murina* organisms treated with EVs containing pSS2.1-Cas9-HH-gRNA*^Dhps^*-HDV and antisense (AS) or sense (S) ssDNA. Donor DNA for Dhps^ARS^ is displayed in bottom row of alignment. Treated Pm organisms all display precise genetic editing to include the associated nucleotide changes for an amino acid change from TRP to ARS, as well as silent point mutations included on the donor DNA. TX, TMX-SMX groups are shown. Red indicates change in sequence from DHPS^WT^ isolated from Pm.

For *in vivo Dhps* editing, we engineered a Cas9 expression cassette encoding a guide RNA targeting the *Dhps* locus, flanked by a hammerhead (HH) ribozyme at the 5′ end and a hepatitis delta virus (HDV) ribozyme at the 3′ end (Fig. 5A). This ribozyme-flanked gRNA design was inserted into the CDS region of plasmid pSS2.1 and is based on a previously described self-processing architecture (24). In this system, the HH and HDV ribozymes mediate RNA self-cleavage (25, 26), resulting in the generation of mature *Cas9* mRNA and *Dhps*-targeting gRNA. The resulting construct (pSS2.1-*Cas9-HH-gRNA*^*Dhps*^-*HDV*) was delivered to *P. murina* via EVs, along with either sense or antisense single-stranded DNA (ssDNA) donor templates encoding the *Dhps*^*ARS*^ mutation. Both orientations of the donor were tested to account for potential strand bias during homology-directed repair (HDR).

Following *in vitro* blasticidin selection to enrich for transformed organisms, mice were infected with the treated *P. murina* organisms. After 5 weeks post-inoculation, the animals were treated with TMP-SMX for 2 weeks. This treatment regimen applied strong selective pressure, first with blasticidin, for which resistance is encoded on the plasmid, and then with TMP-SMX, where only *Pneumocystis* organisms that had successfully incorporated the *Dhps*^*ARS*^ mutation were resistant to SMX exposure (9–13).

After 5 weeks of infection and 2 weeks of treatment, quantitative PCR revealed that *Dhps^ARS^* copy number was detectable in both donor-treated groups compared to *Dhps*^*WT*^, which was not detected (Fig. 5B). These results suggest that delivery of either sense or antisense donor templates promoted maintenance or enrichment of the Dhps locus under selective pressure, consistent with successful *in vivo* editing and expansion of resistant organisms. However, treatment with TMP-SMX did not result in a significant decrease in fungal organisms. However, it is plausible that blasticidin selective pressure was sufficient in maintaining only *P. murina* organisms that successfully received pSS2.1 and the encoded Bsd resistance.

To confirm this hypothesis, Sanger sequencing was performed on amplified *Dhps* genomic DNA to provide definitive evidence of precise genetic editing. Primers were designed to amplify regions flanking the donor DNA, thereby eliminating the possibility of amplifying residual plasmid or unincorporated donor template. Alignment of the *Dhps* [1493–1633] region from untreated organisms (*P. murina Dhps^WT^*) was compared to sequences from *P. murina* organisms treated with EVs containing pSS2.1-*Cas9-HH-gRNADhps-HDV* and either antisense (AS) or sense (S) single-stranded DNA (ssDNA) donor templates. The alignment (Fig. 5C) revealed that 10 out of 10 treated animals harbored fungal organisms with successful HDR, displaying precise genomic integration of the *Dhps^ARS^* mutant sequence. In addition to the TRP-to-ARS amino acid substitution, the edited sequences contained silent point mutations engineered into the donor DNA to permit specific quantitative PCR (qPCR) detection and reduce the likelihood of repeated Cas9 ribonucleoprotein (RNP) binding. Together, these findings provide evidence of accurate and durable editing of the *P. murina* genome *in vivo*.

## DISCUSSION

For decades, the inability to culture *Pneumocystis* species *in vitro* has been the principal obstacle to dissecting their biology, metabolism, and pathogenic mechanisms (5). While several studies have reported progress toward axenic or semi-axenic cultivation of *Pneumocystis* spp., including the use of differentiated CuFi-8 airway epithelial cells (27) or axenic media formulations (28, 29), these approaches have not been widely adopted, in part due to challenges in reproducibility across research groups. In addition, efforts to apply alternative gene delivery strategies, including lipid nanoparticles and electroporation, were unsuccessful in our hands, though these methods may warrant further optimization.

These major bottlenecks have severely limited the application of modern molecular genetics to this clinically important pathogen. Here, we report an EV-mediated system that bypasses these limitations and establishes the first robust *in vivo* method for genetic manipulation of *Pneumocystis murina*. Because the fungus appears to internalize host EVs naturally, potentially for nutrient acquisition (20), our strategy exploits an existing uptake pathway, which likely underlies the high transformation and transgene-expression efficiencies we observed.

We selected a *P. murina*-infected mouse model because the extensive availability of genetically engineered mouse strains allows for mechanistic host–pathogen studies that are not readily available in other systems. This genetic flexibility enables experimental designs that combine precise pathogen modifications with targeted host gene alterations. For example, a *P. murina* strain engineered to overexpress genes involved in β-glucan synthesis and deposition could be used to infect mice lacking the β-glucan receptor dectin-1, thereby allowing the hypothesis that host receptor deficiency attenuates β-glucan-driven inflammatory responses *in vivo* to be tested.

Our study introduces two distinct yet complementary genetic manipulation platforms. The first is a plasmid-based system designed for stable gene overexpression and complementation studies. To achieve sustained gene expression beyond transient delivery, the pSS2.1 plasmid carried a blasticidin-resistance cassette for initial enrichment and a truncated *PmCen15* centromeric region designed to promote episomal maintenance. Consistent with this design, we detected transgene expression for at least 5 weeks *in vivo*. Although plasmid retention was not directly quantified, consistent mRNA and protein detection across multiple time points supports stable maintenance. These data suggest that inclusion of the centromeric element significantly reduced plasmid loss, thereby permitting longitudinal analyses of gene function and host–pathogen interactions under physiologically relevant conditions. As a plasmid-based expression system, pSS2.1 thus provides a rapid platform for gene overexpression or complementation experiments without permanent genomic modification, enabling researchers to study the effects of increased protein levels or restore gene function in mutant strains. This stable maintenance *in vivo* is a major methodological achievement, opening up new possibilities for conducting prolonged functional studies and investigating drug efficacy over time in a physiologically relevant setting.

Building on that stable plasmid platform, we developed and demonstrated a high-fidelity CRISPR/Cas9 system for precise HDR of the *Dhps* gene. This system utilized a *Cas9* expression cassette with a guide RNA flanked by hammerhead and HDV ribozymes, following the self-processing gRNA architecture described by Gao and Zhao (24). The successful *in vitro* cleavage confirmed the functionality of our designed crRNAs and Cas9 enzyme. The subsequent *in vivo* application yielded remarkable results: all 10 *P. murina* populations, each derived from independently infected mice, carried the Dhps^ARS^ mutation, confirmed by Sanger sequencing. This high efficiency of precise HDR *in vivo* is a groundbreaking achievement for an obligate pathogen. The successful demonstration of HDR in all 10 *P. murina* populations is significant, particularly given the general challenges associated with HDR efficiency (30, 31) and the extreme difficulty of genetically manipulating *Pneumocystis*. This high efficiency of precise HDR is particularly notable, given that *Pneumocystis* appears to primarily rely on HR for DNA repair, potentially lacking non-homologous end-joining pathways (32). This suggests that our system successfully directed the organism’s inherent HR machinery for precise genomic modification. This efficiency, coupled with the precision of HDR (30, 31), means that specific, predetermined genetic changes can now be reliably introduced into *P. murina in vivo*.

Blasticidin selection *in vitro* was sufficient to enrich for initial plasmid uptake, while subsequent *in vivo* trimethoprim/sulfamethoxazole treatment confirmed complete resistance in the edited fungi. This second drug pressure provided clear phenotypic validation of the rare, precise editing events, offering a reliable route to identify drug-resistant, site-specific mutants. The ability to precisely introduce the *Dhps*^*ARS*^ mutation, a clinically relevant resistance determinant in *P. jirovecii* (9–13), directly demonstrates the power of this CRISPR platform. This system complements the plasmid-based approach by enabling stable knock-in, knock-out, or point-mutation studies at native chromosomal loci, allowing for investigations into essential gene functions, protein localization, and the molecular mechanisms of drug resistance.

Together, the stable EV-mediated plasmid transformation for overexpression and the high-fidelity CRISPR editing for precise genome modification furnish complementary tools that finally open *Pneumocystis* research to sophisticated molecular genetics. Our *in vivo Dhps*^*ARS*^ model now enables direct, empirical testing of antifolate compounds previously assessed only in heterologous species (33, 34), allowing determination of resistance thresholds, binding efficacy, and pharmacodynamic performance in the natural pulmonary environment. This EV-based system can be readily adapted for functional genomic interventions. Investigators can now generate specific drug-resistant strains for *in vivo* drug screening, tag endogenous proteins for localization and interaction studies, or create conditional knockouts of essential genes. These approaches were previously inaccessible for this pathogen. These capabilities are vital for understanding *Pneumocystis* pathogenesis, identifying novel drug targets, and ultimately developing more effective therapies for PjP, especially in the face of emerging drug resistance.

However, the current system has limitations, including the relatively prolonged timeline required. Each genetically edited *P. murina* population necessitates an initial mouse infection lasting approximately 5–6 weeks to yield ~10^4^ organisms, which then require further propagation in another mouse for an additional 5–6 weeks to achieve larger populations. Additionally, dependence on animal models inherently constrains throughput and scalability compared to traditional *in vitro* cultivation methods. Nonetheless, these limitations are expected to be addressed when a suitable *in vitro* culture system for *Pneumocystis* is developed, which would facilitate selection, scalability, and throughput.

Although the current system requires a prolonged *in vivo* propagation cycle and is constrained by dependence on animal models, its underlying principles are broadly adaptable. Beyond *P. murina*, this strategy holds promise for other genetically inaccessible pathogens. *Mycobacterium leprae*, the leprosy bacillus, remains unculturable in broth media and depends entirely on host tissues for replication (35, 36). Its close relative, *Mycobacterium lepromatosis*, shares this obligate dependency due to extensive genome reduction.

In addition, many human pathogens can enter a viable but non-culturable state, remaining metabolically active but unable to form colonies on standard media (37). These include *Mycobacterium tuberculosis*, *Helicobacter pylori*, *Legionella pneumophila*, *Pseudomonas aeruginosa*, and *Vibrio vulnificus*. For pathogens that naturally internalize host-derived EVs, our platform could offer a route to genetic manipulation where culture-based methods fail. Preliminary EV uptake assays, such as with fluorescently labeled EVs, would be an essential first step in identifying suitable candidates for adaptation of this technology.

This work delivers a groundbreaking genetic toolkit for *P. murina*, fundamentally transforming the *Pneumocystis* research landscape. These strategies are potentially applicable to other unculturable obligate pathogens facing similar genetic manipulation challenges. By overcoming the long-standing barriers of genetic intractability, this platform significantly advances our ability to investigate *Pneumocystis* biology and pathogenicity at an unprecedented level of molecular detail. By enabling both durable transgene expression and highly efficient HDR *in vivo*, this toolkit paves the way for comprehensive mechanistic studies, precise genetic engineering, and accelerated drug discovery, fundamentally enhancing our capacity to combat *Pneumocystis* pneumonia.

## MATERIALS AND METHODS

### Animals and *P. murina* collection

BALB/c male mice (20–25 g, 5–6 weeks old) were immunosuppressed for the duration of the study with dexamethasone (4 mg/L) in drinking water, provided *ad libitum*. Mice were infected via intranasal inoculation with 10^6^ *P. murina*, cryopreserved from previously infected mice. The inoculum was administered under isoflurane anesthesia. Mice were euthanized humanely, and their lungs removed for isolation and quantification of fungal organisms (*n* = 5). To quantify fungal burden, lungs were homogenized in phosphate buffered saline (PBS) using gentleMACS (Miltenyi Biotec, Auburn, CA, USA). The lung homogenate was sequentially filtered through 70 µm then 30 µm filters. The resulting filtrate was then centrifuged at 300 × *g* for 10 min to pellet host cells. The supernatant was then pelleted at 3,500 × *g* for 10 min and resuspended in 10 mL. Samples were placed onto a slide, heat fixed, then stained with a modified Diff-Quik staining to visualize the nuclei for microscopic enumeration, as previously described in Cushion et al. (38).

### EV isolation and characterization

The mouse lung EVs used in this study are those isolated in Sayson et al. (20). Briefly, BALF was collected from uninfected, immunocompetent BALB/c mice using cold PBS (1 mL × 3), and the cellular debris was removed by centrifugation at 3,400 × *g* for 15 min. Size exclusion chromatography was performed on BALF using qEV10 columns and the Automatic Fraction Collector (Izon Science, Medford, MA, USA) to isolate EVs from fractions 1–5. EVs were quantified using the Micro BCA Protein Assay Kit (Thermo Scientific, Rockford, IL, USA). Quality control measures were performed as previously described (20), which included nanoparticle tracking analysis for size distribution and concentration, electron microscopy for morphological assessment, and Western blot analysis for common EV markers (e.g., CD9, CD63, TSG101) and absence of cellular contaminants (data not shown; see reference 20).

### *Pneumocystis murina* maintenance cultures and isolation

Cryopreserved *P. murina* organisms from infected mice were thawed briefly at 37°C, then resuspended in Roswell Park Memorial Institute (RPMI) 1640 medium. Organisms were pelleted at 300 × *g* for 10 min, and the resulting pellet was resuspended at a density of 2 × 10^6^ cells/mL using RPMI 1640 medium (Gibco, Grand Island, NY, USA) supplemented with 10% fetal bovine serum (Cytiva, Marlborough, MA, USA), 1,000 U/mL penicillin, 1,000 µg/mL streptomycin (Gibco, Grand Island, NY, USA), 1% minimum essential medium (MEM) vitamin solution (Gibco, Grand Island, NY, USA), and 1% MEM non-essential amino acid solution (Gibco, Grand Island, NY, USA) (39, 40). Short-term maintenance cultures were set up by distributing 10^6^ *P. murina* organisms in triplicate into 48-well polystyrene plates (Corning Costar, Glendale, AZ, USA) and incubated at 37°C in a 5% CO_2_ atmosphere for an initial 2 h before EV treatment, as described below.

### Plasmid backbone construction

The initial pSS1 plasmid was designed and constructed from gene fragments. The pSS1 plasmid (Fig. 2A) was engineered for the transfer and expression of genetic material, encoding either *Dhps^ARS^* or mNeonGreen. These genes were under the transcriptional control of a *P. murina Msg* promoter, which has been previously shown to exhibit strong transcriptional activity in heterologous systems, including *Saccharomyces cerevisiae* (41, 42). A 151 bp sequence downstream of the 3′ untranslated region of *Namp8* (*PNEG_1673*) was selected and used as a transcriptional terminator.

Subsequent modifications and the generation of various derivative versions, including pSS2.1, were designed in-house and constructed by VectorBuilder (www.vectorbuilder.com). The pSS2.1 plasmid (Fig. 3A) was further enhanced by the incorporation of a blasticidin resistance gene, *Bsd*, for selection. This was under transcriptional control of a *Tef1* (*PNEG_01204*) promoter (444 bp upstream of start codon) and terminator (500 bp downstream of stop codon). Additionally, a truncated PmCen15 centromeric region was added to ensure stable maintenance (23).

To promote efficient transgene expression, the coding sequences for *Bsd*, *mNeonGreen*, and *Cas9* were codon-optimized for *P. murina* codon usage using the online codon optimization tool provided by NovoPro (https://www.novoprolabs.com/tools/codon-optimization).

### EV transformation and *in vitro* delivery to *P. murina*

Plasmids and siRNA were transformed into purified mouse lung EVs utilizing the Exo-Fect Exosome Transfection Kit, following the manufacturer’s protocol (System Biosciences, Palo Alto, CA, USA). Transformation reactions were stopped, and EVs were purified using ExoQuick-TC (System Biosciences, Palo Alto, CA, USA) to remove residual nucleic acids, then resuspended in PBS. A 2 µg protein equivalent of loaded EVs or control EVs was introduced to 10^6^ *P. murina* and incubated at 37°C in a 5% CO_2_ atmosphere for a 24 h period, as described above.

### Fluorescent microscopy

Cells were fixed in 3.7% formaldehyde prepared in PBS for 15 min following a PBS wash. After fixation, cells were transferred onto microscope slides using a CytoSpin 2 cytocentrifuge (Thermo Shandon, Kalamazoo, MI, USA) at 1,000 rpm for 10 min. Non-specific binding was blocked by incubating samples in 10% goat serum for 1 h. Cells were then stained with anti-Msg primary antibodies, followed by Alexa Fluor 488-conjugated anti-rabbit secondary antibodies, each applied for 1 h. Between antibody incubations, samples were washed three times for 15 min each with PBS containing 0.1% Tween-20. Imaging was performed on an Olympus IX83 inverted fluorescence microscope.

### RNA and DNA isolation

Total RNA was extracted from *P. murina* cultures utilizing the ZymoResearch Direct-zol RNA Miniprep Kit (Zymo Research, Irvine, CA, USA) and treated with DNase I (Zymo Research, Irvine, CA, USA), according to the manufacturer’s instructions. Genomic DNA was extracted from *P. murina* organisms isolated from lung samples using the ZymoResearch Quick-DNA Miniprep Kit (Zymo Research, Irvine, CA, USA).

### RT-qPCR and qPCR

cDNA was synthesized from isolated RNA using SuperScript IV VILO Master Mix (Invitrogen, Carlsbad, CA, USA). RT-qPCR was performed on mNeonGreen expression normalized against large subunit (LSU) rRNA using PowerUp SYBR Green Master Mix (Applied Biosystems, Waltham, MA, USA). RT-qPCR was performed on *Dhps*^*WT*^ or *Dhps*^*ARS*^ expression normalized against LSU using TaqMan Fast Advanced Master Mix (Applied Biosystems, Waltham, MA, USA). Real-time PCR assays were run on an Applied Biosciences 7500 Fast PCR system. Relative expression was calculated using the 2^−ΔCt^ method, normalized to the endogenous housekeeping gene *LSU*.

For genomic DNA copy number analysis, qPCR was performed using TaqMan Fast Advanced Master Mix. A standard curve was generated using synthetic gene fragments (Azenta Life Sciences, South Plainfield, NJ, USA) of *Dhps*^*WT*^ and *Dhps*^*ARS*^ to determine absolute copy numbers. Primer, probe, and gene fragment sequences are provided in Table S1.

### ELISA

The presence of mNeonGreen *in vivo* was detected by ELISA. Lung homogenates were lysed in radioimmunoprecipitation assay buffer (RIPA) buffer (Alfa Aesar, Ward Hill, MA, USA). High-binding 96-well plates (Corning 2592; Kennebunk, ME, USA) were coated overnight with capture antibody, mNeonGreen VHH Recombinant Alpaca Monoclonal Antibody (ChromoTek CTK0203; Planegg-Martinsried, Germany), in 50 mM carbonate buffer, pH 9.4, at 4°C. Lung lysates were diluted 1:10 in PBS and then added to the plate for 1 h. After antigen capture, wells were washed three times with PBS with 0.1% Tween-20 (PBST), then detected using mNeonGreen polyclonal antibody (Proteintech 29523-1-AP, Planegg-Martinsried, Germany) and goat anti-rabbit IgG (H + L) secondary antibody, HRP (Invitrogen 31460, Waltham, MA, USA). After washing with PBST three times, detection was performed using 1-Step TMB ELISA Substrate Solutions (Thermo Scientific, Rockford, IL, USA), and absorbance at 650 nm was read using a Synergy HTX plate reader (BioTek, Winooski, VT, USA).

### CRISPR reagents and *in vitro* cleavage assay

Two distinct sets of crRNA targeting the *P. murina Dhps* gene were designed using the CRISPR tool in Benchling (www.benchling.com). *P. murina Dhps* was amplified by PCR from gDNA and used for cleavage assays. CRISPR RNA (crRNA) was annealed to trans-activating CRISPR RNA (tracrRNA) (IDT, Coralville, IA, USA) at a 1:1 ratio to form a guide RNA (gRNA), then complexed with recombinant Alt-R *S.p.* Cas9 protein (IDT, Coralville, IA, USA). *In vitro* cleavage assays were conducted by incubating gRNA:Cas9 RNP complexes with *Dhps^WT^* DNA for 60 min at 37°C. After the digestion was completed, Proteinase K (1.81 mg; Zymo Research, Irvine, CA, USA) was added to stop the reaction. Cleavage products were resolved on a 1% agarose gel in TAE buffer, then stained with GelRed Nucleic Acid Gel Stain (Biotium, Fremont, CA, USA). Gels were visualized and analyzed on an iBright CL1500 imaging system (Invitrogen, Carlsbad, CA, USA). Densitometry analysis was performed employing the iBright software to quantify cleavage efficiency. The specific crRNA sequences and PCR primers are provided in Table S1.

### *In vivo* experimental design

*P. murina* (1 × 10^6^) was co-cultured with either untreated EVs or transformed EVs, as described above. After 2 h of incubation, to allow expression of Bsd, blasticidin (100 µg/mL; Gibco, Grand Island, NY, USA) selection was applied *in vitro* for 24 h. *P. murina* was then washed three times in PBS and intranasally inoculated into mice. For pSS2.1-mNeonGreen experiments, lung samples were collected at 1 week, 3 weeks, and 5 weeks post-inoculation for subsequent analysis. For CRISPR/Cas9 experiments, mice were allowed to develop infection for 5 weeks, followed by oral gavage with Sulfamethoxazole and Trimethoprim Oral Suspension (125 mg/kg, 12.5 mg/kg; Aurobindo Pharma USA, East Windsor, NJ, USA) three times a week for 2 weeks (43). Lung samples were collected at the end of the 2-week treatment period.

### Sanger sequencing

Genomic DNA was extracted from *P. murina* isolated from mouse lungs and amplified using primers positioned outside the donor DNA regions to ensure amplification of genomic DNA rather than residual donor DNA. The amplified products were submitted to Genewiz (www.genewiz.com) for Sanger sequencing. Sequencing primer is provided in Table S1. Sequence alignments were performed using Clustal Omega to confirm successful homologous recombination (44).

### Statistical analysis

Statistical analysis was performed in GraphPad Prism version 10.4.2 (534) using unpaired *t*-tests or two-way analysis of variance followed by Sidak’s multiple comparisons *post hoc* test to control groups. A *P* value <0.05 was considered statistically significant.

## SUPPLEMENTAL MATERIAL

Table S1 mbio.01825-25-s0001.pdf Primer, crRNA, gene fragment, and dDNA sequences.

ASM does not own the copyrights to Supplemental Material that may be linked to, or accessed through, an article. The authors have granted ASM a non-exclusive, world-wide license to publish the Supplemental Material files. Please contact the corresponding author directly for reuse.

## References

[1] Kanj A, Samhouri B, Abdallah N, Chehab O, Baqir M. 2021. Host factors and outcomes in hospitalizations for Pneumocystis jirovecii pneumonia in the United States. Mayo Clin Proc 96:400–407. doi:10.1016/j.mayocp.2020.07.029 33549258

[2] Kolbrink B, Scheikholeslami-Sabzewari J, Borzikowsky C, von Samson-Himmelstjerna FA, Ullmann AJ, Kunzendorf U, Schulte K. 2022. Evolving epidemiology of Pneumocystis pneumonia: findings from a longitudinal population-based study and a retrospective multi-center study in Germany. Lancet Reg Health Eur 18:100400. doi:10.1016/j.lanepe.2022.100400 35814339 PMC9257643

[3] Brown GD, Denning DW, Gow NAR, Levitz SM, Netea MG, White TC. 2012. Hidden killers: human fungal infections. Sci Transl Med 4:165rv113. doi:10.1126/scitranslmed.3004404 23253612

[4] Xue T, Kong X, Ma L. 2023. Trends in the epidemiology of Pneumocystis pneumonia in immunocompromised patients without HIV infection. J Fungi (Basel) 9:812. doi:10.3390/jof9080812 37623583 PMC10455156

[5] Cushion MT, Tisdale-Macioce N, Sayson SG, Porollo A. 2021. The persistent challenge of Pneumocystis growth outside the mammalian lung: past and future approaches. Front Microbiol 12:681474. doi:10.3389/fmicb.2021.681474 34093506 PMC8174303

[6] Hauser PM, Burdet FX, Cissé OH, Keller L, Taffé P, Sanglard D, Pagni M. 2010. Comparative genomics suggests that the fungal pathogen Pneumocystis is an obligate parasite scavenging amino acids from its host’s lungs. PLoS One 5:e15152. doi:10.1371/journal.pone.0015152 21188143 PMC3004796

[7] Hughes WT, Feldman S, Sanyal SK. 1975. Treatment of Pneumocystis carinii pneumonitis with trimethoprim-sulfamethoxazole. Can Med Assoc J 112:47–50. 1079469 PMC1956451

[8] Hughes WT, Feldman S, Chaudhary SC, Ossi MJ, Cox F, Sanyal SK. 1978. Comparison of pentamidine isethionate and trimethoprim-sulfamethoxazole in the treatment of Pneumocystis carinii pneumonia. J Pediatr 92:285–291. doi:10.1016/s0022-3476(78)80028-6 304478

[9] Kazanjian P, Locke AB, Hossler PA, Lane BR, Bartlett MS, Smith JW, Cannon M, Meshnick SR. 1998. Pneumocystis carinii mutations associated with sulfa and sulfone prophylaxis failures in AIDS patients. AIDS 12:873–878. doi:10.1097/00002030-199808000-00009 9631140

[10] Armstrong W, Meshnick S, Kazanjian P. 2000. Pneumocystis carinii mutations associated with sulfa and sulfone prophylaxis failures in immunocompromised patients. Microbes Infect 2:61–67. doi:10.1016/s1286-4579(00)00284-7 10717542

[11] Zingale A, Carrera P, Lazzarin A, Scarpellini P. 2003. Detection of Pneumocystis carinii and characterization of mutations associated with sulfa resistance in bronchoalveolar lavage samples from human immunodeficiency virus-infected subjects. J Clin Microbiol 41:2709–2712. doi:10.1128/JCM.41.6.2709-2712.2003 12791912 PMC156552

[12] Lee K, Lee WG, Uh Y, Ha GY, Cho J, Chong Y, Korean Nationwide Surveillance of Antimicrobial Resistance Group. 2003. VIM- and IMP-type metallo-beta-lactamase-producing Pseudomonas spp. and Acinetobacter spp. in Korean hospitals. Emerg Infect Dis 9:868–871. doi:10.3201/eid0907.020753 12890331 PMC3023439

[13] Meneau I, Sanglard D, Bille J, Hauser PM. 2004. Pneumocystis jiroveci dihydropteroate synthase polymorphisms confer resistance to sulfadoxine and sulfanilamide in Saccharomyces cerevisiae. Antimicrob Agents Chemother 48:2610–2616. doi:10.1128/AAC.48.7.2610-2616.2004 15215117 PMC434158

[14] Keely SP, Renauld H, Wakefield AE, Cushion MT, Smulian AG, Fosker N, Fraser A, Harris D, Murphy L, Price C, Quail MA, Seeger K, Sharp S, Tindal CJ, Warren T, Zuiderwijk E, Barrell BG, Stringer JR, Hall N. 2005. Gene arrays at Pneumocystis carinii telomeres. Genetics 170:1589–1600. doi:10.1534/genetics.105.040733 15965256 PMC1449779

[15] Ma L, Chen Z, Huang DW, Kutty G, Ishihara M, Wang H, Abouelleil A, Bishop L, Davey E, Deng R, et al.. 2016. Genome analysis of three Pneumocystis species reveals adaptation mechanisms to life exclusively in mammalian hosts. Nat Commun 7:10740. doi:10.1038/ncomms10740 26899007 PMC4764891

[16] Ma L, Chen Z, Huang DW, Cissé OH, Rothenburger JL, Latinne A, Bishop L, Blair R, Brenchley JM, Chabé M, et al.. 2020. Diversity and complexity of the large surface protein family in the compacted genomes of multiple Pneumocystis Species. mBio 11:e02878-19. doi:10.1128/mBio.02878-19 32127451 PMC7064768

[17] Kutty G, Maldarelli F, Achaz G, Kovacs JA. 2008. Variation in the major surface glycoprotein genes in Pneumocystis jirovecii. J Infect Dis 198:741–749. doi:10.1086/590433 18627244 PMC2561252

[18] Meier CS, Pagni M, Richard S, Mühlethaler K, Hauser PM. 2025. Selective expression of Pneumocystis antigens in different patients during a suspected outbreak of Pneumocystis pneumonia. mBio 16:e00692-25. doi:10.1128/mbio.00692-25 40243367 PMC12077189

[19] Javeed N, Mukhopadhyay D. 2017. Exosomes and their role in the micro-/macro-environment: a comprehensive review. J Biomed Res 31:386–394. doi:10.7555/JBR.30.20150162 28290182 PMC5706431

[20] Sayson SG, Ashbaugh A, Cushion MT. 2024. Extracellular vesicles from Pneumocystis carinii-infected rats impair fungal viability but are dispensable for macrophage functions. Microbiol Spectr 12:e03653-23. doi:10.1128/spectrum.03653-23 38236033 PMC10845964

[21] Ran FA, Hsu PD, Wright J, Agarwala V, Scott DA, Zhang F. 2013. Genome engineering using the CRISPR-Cas9 system. Nat Protoc 8:2281–2308. doi:10.1038/nprot.2013.143 24157548 PMC3969860

[22] Li T, Yang Y, Qi H, Cui W, Zhang L, Fu X, He X, Liu M, Li PF, Yu T. 2023. CRISPR/Cas9 therapeutics: progress and prospects. Signal Transduct Target Ther 8:36. doi:10.1038/s41392-023-01309-7 36646687 PMC9841506

[23] Cissé OH, Curran SJ, Folco HD, Liu Y, Bishop L, Wang H, Fischer ER, Davis AS, Combs C, Thapar S, Dekker JP, Grewal S, Cushion M, Ma L, Kovacs JA. 2024. Regional centromere configuration in the fungal pathogens of the Pneumocystis genus. mBio 15:e03185-23. doi:10.1128/mbio.03185-23 38380929 PMC10936427

[24] Gao Y, Zhao Y. 2014. Self-processing of ribozyme-flanked RNAs into guide RNAs in vitro and in vivo for CRISPR-mediated genome editing. J Integr Plant Biol 56:343–349. doi:10.1111/jipb.12152 24373158

[25] Pley HW, Flaherty KM, McKay DB. 1994. Three-dimensional structure of a hammerhead ribozyme. Nature 372:68–74. doi:10.1038/372068a0 7969422

[26] Ferré-D’Amaré AR, Zhou K, Doudna JA. 1998. Crystal structure of a hepatitis delta virus ribozyme. Nature 395:567–574. doi:10.1038/26912 9783582

[27] Schildgen V, Mai S, Khalfaoui S, Lüsebrink J, Pieper M, Tillmann RL, Brockmann M, Schildgen O. 2014. Pneumocystis jirovecii can be productively cultured in differentiated CuFi-8 airway cells. mBio 5:e01186-14. doi:10.1128/mBio.01186-14 24825015 PMC4030487

[28] Merali S, Frevert U, Williams JH, Chin K, Bryan R, Clarkson AB Jr. 1999. Continuous axenic cultivation of Pneumocystis carinii. Proc Natl Acad Sci USA 96:2402–2407. doi:10.1073/pnas.96.5.2402 10051654 PMC26796

[29] Riebold D, Mahnkopf M, Wicht K, Zubiria-Barrera C, Heise J, Frank M, Misch D, Bauer T, Stocker H, Slevogt H. 2023. Axenic long-term cultivation of Pneumocystis jirovecii. J Fungi (Basel) 9:903. doi:10.3390/jof9090903 37755011 PMC10533121

[30] Liao H, Wu J, VanDusen NJ, Li Y, Zheng Y. 2024. CRISPR-Cas9-mediated homology-directed repair for precise gene editing. Mol Ther Nucleic Acids 35:102344. doi:10.1016/j.omtn.2024.102344 39494147 PMC11531618

[31] Miyaoka Y, Berman JR, Cooper SB, Mayerl SJ, Chan AH, Zhang B, Karlin-Neumann GA, Conklin BR. 2016. Systematic quantification of HDR and NHEJ reveals effects of locus, nuclease, and cell type on genome-editing. Sci Rep 6:23549. doi:10.1038/srep23549 27030102 PMC4814844

[32] Cisse OH, Ma L, Kovacs JA. 2024. Retracing the evolution of Pneumocystis species, with a focus on the human pathogen Pneumocystis jirovecii. Microbiol Mol Biol Rev 88:e00202-22. doi:10.1128/mmbr.00202-22 38587383 PMC11332345

[33] Iliades P, Meshnick SR, Macreadie IG. 2004. Dihydropteroate synthase mutations in Pneumocystis jiroveci can affect sulfamethoxazole resistance in a Saccharomyces cerevisiae model. Antimicrob Agents Chemother 48:2617–2623. doi:10.1128/AAC.48.7.2617-2623.2004 15215118 PMC434176

[34] Iliades P, Meshnick SR, Macreadie IG. 2005. Mutations in the Pneumocystis jirovecii DHPS gene confer cross-resistance to sulfa drugs. Antimicrob Agents Chemother 49:741–748. doi:10.1128/AAC.49.2.741-748.2005 15673759 PMC547354

[35] Pattyn SR. 1973. The problem of cultivation of Mycobacterium leprae. Bull World Health Organ 49:403–410. 4212439 PMC2480944

[36] de Paula NA, Leite MN, de Faria Bertoluci DF, Soares CT, Rosa PS, Frade MAC. 2024. Human skin as an ex vivo model for maintaining Mycobacterium leprae and leprosy studies. Trop Med Infect Dis 9:135. doi:10.3390/tropicalmed9060135 38922047 PMC11209558

[37] Pazos-Rojas LA, Cuellar-Sánchez A, Romero-Cerón AL, Rivera-Urbalejo A, Van Dillewijn P, Luna-Vital DA, Muñoz-Rojas J, Morales-García YE, Bustillos-Cristales MDR. 2023. The viable but non-culturable (VBNC) state, a poorly explored aspect of beneficial bacteria. Microorganisms 12:39. doi:10.3390/microorganisms12010039 38257865 PMC10818521

[38] Cushion MT, Ashbaugh A, Hendrix K, Linke MJ, Tisdale N, Sayson SG, Porollo A. 2018. Gene expression of Pneumocystis murina after treatment with anidulafungin results in strong signals for sexual reproduction, cell wall integrity, and cell cycle arrest, indicating a requirement for ascus formation for proliferation. Antimicrob Agents Chemother 62:e02513-17. doi:10.1128/AAC.02513-17 29463544 PMC5923105

[39] Collins MS, Cushion MT. 2001. Standardization of an in vitro drug screening assay by use of cryopreserved and characterized Pneumocystis carinii populations. J Eukaryot Microbiol Suppl:178S–179S. doi:10.1111/j.1550-7408.2001.tb00509.x 11906052

[40] Porollo A, Sayson SG, Ashbaugh A, Rebholz S, Landero Figueroa JA, Cushion MT. 2024. Insights into copper sensing and tolerance in Pneumocystis species. Front Microbiol 15:1383737. doi:10.3389/fmicb.2024.1383737 38812685 PMC11133566

[41] Kutty G, Shroff R, Kovacs JA. 2013. Characterization of Pneumocystis major surface glycoprotein gene (msg) promoter activity in Saccharomyces cerevisiae. Eukaryot Cell 12:1349–1355. doi:10.1128/EC.00122-13 23893080 PMC3811333

[42] Broomall K, Collins M, Smulian AG. 1997. Pneumocystis carinii promoter analysis in a heterologous Saccharomyces cerevisiae assay system. J Eukaryot Microbiol 44:10S–11S. doi:10.1111/j.1550-7408.1997.tb05740.x 9508404

[43] Cushion MT, Linke MJ, Ashbaugh A, Sesterhenn T, Collins MS, Lynch K, Brubaker R, Walzer PD. 2010. Echinocandin treatment of Pneumocystis pneumonia in rodent models depletes cysts leaving trophic burdens that cannot transmit the infection. PLoS One 5:e8524. doi:10.1371/journal.pone.0008524 20126455 PMC2813285

[44] Sievers F, Higgins DG. 2018. Clustal Omega for making accurate alignments of many protein sequences. Protein Sci 27:135–145. doi:10.1002/pro.3290 28884485 PMC5734385

