# Supplementary material for: Extracellular vesicle-mediated delivery of genetic material for transformation and CRISPR/Cas9-based gene editing in Pneumocystis murina
Source: mBio. 2025 Sep 23;16(11):e01825-25. doi: 10.1128/mbio.01825-25 (PMC12607567; doi:10.1128/mbio.01825-25)
Supplement: Table S1 — Primer, crRNA, gene fragment, and dDNA sequences. [file mbio.01825-25-s0001.pdf]

Supplementary Table 1: Primer, crRNA, gene fragment, and dDNA sequences

**Name**	**Product**	**Sequence**
scramble Control	crRNA	/AlTR1/rCrCrArUrCrArGrCrArCrUrCrGrCrCrArArUrUrGrGrUrUrUrArGrArGrCrUrArUrGrCrU/AlTR2/
Antisense *dhps*	crRNA	/AlTR1/rArArArGrGrUrArUrArArUrArUrGrCrGrArArCrCrGrUrUrUrArGrArGrCrUrArUrGrCrU/AlTR2/
Sense *dhps*	crRNA	/AlTR1/rGrGrUrGrGrGrCrArGrUrCrUrArCrArArGrArCrCrGrUrUrUrArGrArGrCrUrArUrGrCrU/AlTR2/
Dhps-1462F	qPCR primer	GATGGAGGTATTCATTCATGTGAT
Dhps-1614-R	qPCR primer	AGCAGGAATAACTCGAAAAATCTC
dhps TRP probe	internal probe	/56-FAM/TGGGCAGTC+T+ACAAGACCAGGT/3IABkFQ/
dhps ARS probe	internal probe	/5Cy3/AGG+TCAGTC+A+GCAAGA+TCAGGA/3IAbRQSp/
Dhps-1F	primer	ATGATATTTAAAACAAAAAGTTTAAAAATATCTCC
Dhps-2229R	primer	TTAATAAATTTCTTTCCAAATAGCATCTG
Dhps-1350F	sequencing primer	GCCGGTTTTGCCATTCTTAT
LSU-F	qPCR primer	ATGAGGTGAAAAGTCGAAAGGG
LSU-R	qPCR primer	TGATTGTCTCAGATGAAAAACCTCTT
LS-P	internal probe	6FAM-AACAGCCCAGAATAATGAATAAAGTTCCTCAATTGTTAC-TAMRA
dhpsWT	Gene fragment for standard curve in qPCR	GCCATTCTTATATAAAAATAAAAGTATGAATTTTAGTTTCAGGTCTTATAAAGTGCCTACATATATTATGGCTATTTTGAATCTTACACCTGACTCTTTTTTTGATGGAGGTATTCATTCATGTGATTCTGTATTAATAGATGTTAAAAAATTTATAAATGCAGGAGCAACAATAGTTGATATTGGTGGGCAGTCTACAAGACCAGGTTCGCATATTATACCTTTAGAAGAAGAGATTTTTCGAGTTATTCCTGCTATAAAATATCTTCTGAAAACATATCCTGATATTTTAATAAGTATAGATACATTTCGTTCTGAAGTTGCAGAACAGGCAGTTAAGGCTGGTGCCAGTCTTGTTAATGATATAAGTGGTGGAAGGTATGATCCAAAAATGCTTAGTACTGTTGCTAGACTAAAGGTTCCAATATGTATAATGCATATGAGAGGCAA

dhpsARS	Gene fragment for standard curve in qPCR	GCCATTCTTATATAAAAATAAAAGTATGAATTTTAGTTTCAGGTCTTATAAAGTGCCTACAT ATATTATGGCTATTTTGAATCTTACACCTGACTCTTTTTTTGATGGAGGTATTCATTCATGTG ATTCTGTATTAATAGATGTTAAAAAATTTATAAATGCAGGAGCAACAATAGTTGATATTGGA GGTCAGTCAGCAAGATCAGGATCGCATATTATACCTTTAGAAGAAGAGATTTTTCGAGTTA TTCCTGCTATAAAATATCTTCTGAAAACATATCCTGATATTTTAATAAGTATAGATACATTTC GTTCTGAAGTTGCAGAACAGGCAGTTAAGGCTGGTGCCAGTCTTGTTAATGATATAAGTG GTGGAAGGTATGATCCAAAAATGCTTAGTACTGTTGCTAGACTAAAGGTTCCAATATGTAT AATGCATATGAGAGGCAA
dhpsARS + donor	ssDNA	/AlT-R-HDR1/G*A*TGGAGGTATTCATTCATGTGATTCTGTATTAATAGATGTTAAAAAATTTATAAAT GCAGGAGCAACAATAGTTGATATTGGAGGTCAGTCAGCAAGATCAGGATCTCATATAATT CCACTTGAAGAAGAGATTTTTCGAGTTATTCCTGCTATAAAATATCTTCTGAAAACATATCC TGATATT TTAATAAGTATA*G*A/AlT-R-HDR2/
dhpsARS - donor	ssDNA	/AlT-R-HDR1/T*C*TATACTTATTAAAATATCAGGATATGTTTTCAGAAGATATTTTATAGCAGGAATA ACTCGAAAAATCTCTTCTTCAAGTGGAATTATATGAGATCCTGATCTTGCTGACTGACCTC CAATATCAACTATTGTTGCTCCTGCATTTATAAATTTTTTAACATCTATTAATACAGAATCAC ATGAATG AATACCTCCA*T*C/AlT-R-HDR2/
